# Representation of Stimulus Speed and Direction in Vibrissal-Sensitive Regions of the Trigeminal Nuclei: A Comparison of Single Unit and Population Responses

**DOI:** 10.1371/journal.pone.0158399

**Published:** 2016-07-27

**Authors:** Aniket S. Kaloti, Erik C. Johnson, Chris S. Bresee, Stephanie N. Naufel, Matthew G. Perich, Douglas L. Jones, Mitra J. Z. Hartmann

**Affiliations:** 1 Interdepartmental Neuroscience Program, Northwestern University, Evanston, IL, United States of America; 2 Department of Electrical and Computer Engineering, University of Illinois, Urbana, IL, United States of America; 3 Coordinated Science Laboratory, University of Illinois, Urbana, IL, United States of America; 4 Beckman Institute for Advanced Science and Technology, University of Illinois, Urbana, IL, United States of America; 5 Department of Biomedical Engineering, Northwestern University, Evanston, IL, United States of America; 6 Advanced Digital Sciences Center, Illinois at Singapore Pte., Singapore, Singapore; 7 Department of Mechanical Engineering, Northwestern University, Evanston, IL, United States of America; McGill University, CANADA

## Abstract

The rat vibrissal (whisker) system is one of the oldest and most important models for the study of active tactile sensing and sensorimotor integration. It is well established that primary sensory neurons in the trigeminal ganglion respond to deflections of one and only one whisker, and that these neurons are strongly tuned for both the speed and direction of individual whisker deflections. During active whisking behavior, however, multiple whiskers will be deflected simultaneously. Very little is known about how neurons at central levels of the trigeminal pathway integrate direction and speed information across multiple whiskers. In the present work, we investigated speed and direction coding in the trigeminal brainstem nuclei, the first stage of neural processing that exhibits multi-whisker receptive fields. Specifically, we recorded both single-unit spikes and local field potentials from fifteen sites in spinal trigeminal nucleus interpolaris and oralis while systematically varying the speed and direction of coherent whisker deflections delivered across the whisker array. For 12/15 neurons, spike rate was higher when the whisker array was stimulated from caudal to rostral rather than rostral to caudal. In addition, 10/15 neurons exhibited higher firing rates for slower stimulus speeds. Interestingly, using a simple decoding strategy for the local field potentials and spike trains, classification of speed and direction was higher for field potentials than for single unit spike trains, suggesting that the field potential is a robust reflection of population activity. Taken together, these results point to the idea that population responses in these brainstem regions in the awake animal will be strongest during behaviors that stimulate a population of whiskers with a directionally coherent motion.

## Introduction

The rat vibrissal system is one of the most prominent models in neuroscience for the study of active touch and sensorimotor integration. Mechanical signals from the vibrissae are represented in the responses of primary sensory neurons in the trigeminal ganglion, each of which responds to one and only one whisker [1–9]. Signals are then sent to the trigeminal brainstem nuclei, which are the first site of neural response integration [10–22]. A fundamental distinction between the responses of neurons in the trigeminal ganglion and those in the trigeminal nuclei is the emergence of multi-whisker receptive fields in the latter [14–16, 23–27].

It is well established that neurons of the trigeminal ganglion exhibit strong angular tuning [9, 28, 29], meaning that the neural response is stronger depending on the direction in which a single whisker is pushed. During active whisking behavior, however, multiple whiskers will tend to be deflected in a coherent manner across the array. Although each individual whisker might bend to a slightly different angle, each whisking motion will generate an overall direction of deflection across regions of the array. A critical open question, then, is how neurons at central levels in the trigeminal pathway represent and integrate information about stimulus speed and direction present across multiple whiskers.

The spinal trigeminal nuclei (the first structures containing neurons exhibiting multi-whisker receptive fields [30]) have also been shown to contain neurons that exhibit angular tuning [31, 32]. It is still not known, however, how trigeminal nuclei neurons with multi-whisker receptive fields represent movement through the whisker array. The present study was undertaken to begin to understand the integrative properties of neurons in the trigeminal nuclei and the extent to which stimulus speed and direction are reflected in the responses of individual neurons compared to responses of the population. Specifically, the study examines the extent to which multi-whisker receptive field neurons code for stimulus directionality across a larger group of neighboring whiskers, and whether there exist differences in neural responses at different speeds of stimulus contact. To further test the encoding of speed and direction, we also consider the problem of classifying, or decoding, the experimental condition from the local field potentials and spike trains recorded experimentally.

## Materials and Methods

We investigated the integrative properties of neurons in the trigeminal nuclei by stimulating the entire rat whisker array with a vertical brass post mounted on a precise servo motor. The post was swept through the vibrissal array at three different speeds (90°/*s*, 180°/*s*, and 360°/*s*) and in both rostral-caudal and caudal-rostral directions. We recorded both single unit and population activity of multi-whisker receptive field neurons in spinal trigeminal nucleus interpolaris (SpVi) and oralis (SpVo) to quantify responses under these different conditions.

### Surgical procedures

All procedures were approved in advance by Northwestern University’s Animal Care and Use Committee. Six female Sprague-Dawley rats (Charles River Laboratories) weighing 240–330 g were used for these experiments. Anesthesia was initially induced with isofluorane (1–2 ml) to minimize handling of the animal. To ensure access to the whiskers a deep surgical plane of anesthesia was maintained using an intraperitoneal injection of ketamine hydrochloride (75.8 mg/kg), Xylazine HCl (3.78 mg/kg) and Acepromazine Maleate (0.76 mg/kg). Supplementary doses were administered to maintain adequate anesthesia (suppressed toe pinch response). Heart rate was monitored throughout the experiment to ensure the health of the animal. Body temperature was maintained at 37.5°C using a servo-controlled heating pad. A craniotomy was performed to expose the cerebellar surface overlying the spinal trigeminal nuclear complex (≈11–14 mm caudal to bregma and 2–3 mm lateral to the midline of the skull). The cerebellar surface was kept moist with mineral oil throughout the experiment.

### Single unit and field potential recordings

Extracellular recordings were performed with tungsten microelectrodes (2–4 MΩ FHC, Inc., Bowdoin, ME, USA). Electrodes were advanced through the cerebellum using a manually controlled micromanipulator (MM33, Sutter Instruments, Novato, CA, USA). While the electrode was advanced into the trigeminal nuclear complex area, whiskers on the ipsilateral side of the face were stimulated manually to detect whisker sensitive units.

Only multi-whisker-sensitive units were recorded in this study. Once a well-isolated whisker sensitive unit was encountered, the receptive field was determined using manual stimulation with a thin (≈1 mm) wooden probe. Manual stimulation allowed us to locate the principal whisker that elicited the maximum response, as observed on the oscilloscope screen and confirmed by listening to the amplified audio recordings of unit activity. We then stimulated the whiskers surrounding the principal whisker to systematically determine all whiskers belonging to the unit’s receptive field, i.e., all the whiskers that elicited a measurable single unit response to whisker deflection. We did not attempt to classify the strength of response of the adjacent whiskers, as our goal was to examine the integrative properties of multi-whisker receptive field neurons. The smallest receptive field was two whiskers (E1 and E2) and the largest receptive field was ten whiskers (C1, C2, C3, C4; D2, D3, D4; B1, B2, B3).

Electrical signals were amplified and filtered (1–10,000 Hz; AM systems, Carlsborg, WA, USA) and digitized at 40 kHz (National Instruments, Austin, TX, USA). To ensure unit isolation throughout the experiment, we noted the response to manual stimulation of the principal whisker between trials and the consistency of spike shape from trial to trial. During recording, care was taken to ensure good spike isolation, so that at least one clearly distinguishable spike waveform with a good signal-to-noise ratio greater than or equal to three was seen on the oscilloscope.

**Fig 1:**
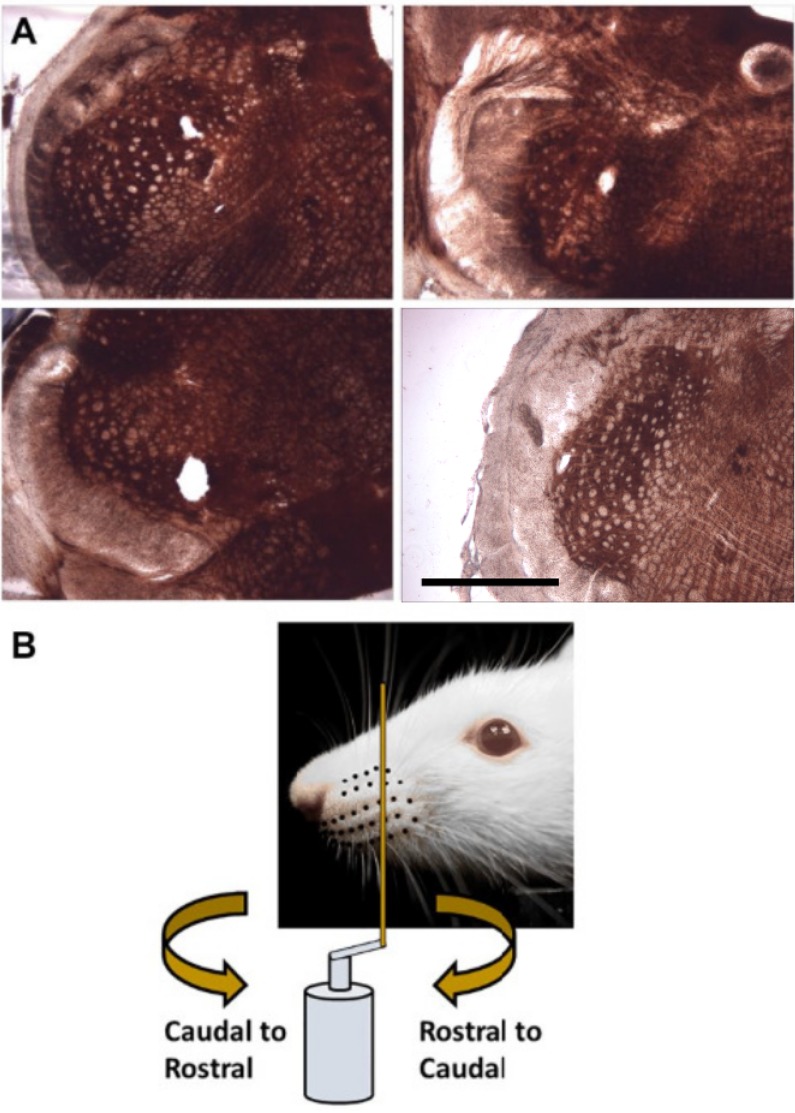
Recording location and whisker stimulation. (A) Histological analysis showed lesions either in spinal trigeminal nucleus interpolaris or spinal trigeminal nucleus oralis. The scale bar represents 1 mm. (B) During stimulation a vertical metal post is mounted on a servo motor and swept through the whisker array in either Rostral-Caudal (RC; clockwise directed curved arrow) or Caudal-Rostral (CR; counter-clockwise directed curved arrow) directions and oriented perpendicular to the whisker array.

After recordings were complete the rat was deeply anesthetized and then euthanized via perfusion with saline and paraformaldehyde, followed by decapitation. The brainstem was removed and sectioned, slide-mounted and stained with cytochrome oxidase using standard histological protocols. Typical coronal sections are shown in Fig 1A.

### Whisker stimulation

All whiskers on both sides of the face were left intact. The left whisker array was stimulated in these experiments using the setup schematized in Fig 1B. A brass metal post (2 mm diameter, 5 cm tall) was vertically mounted on a hard fiber rod (27 cm long, 1/4 inch diameter) attached to the shaft of a servo motor (Animatics, Inc., SM3420, Milpitas, CA, USA) via a custom-made aluminum fitting. The post was swept through the whisker array at three different speeds (90°/*s*, 180°/*s* and 360°/*s*) in Rostral-Caudal (RC, clockwise directed curved arrow) and Caudal-Rostral (CR, counter-clockwise directed curved arrow) directions at an orientation perpendicular to the rows of the whisker array (Fig 1B). This stimulation resulted in linear velocities of 42 cm/s, 85 cm/s, and 170 cm/s, respectively for the corresponding angular speeds.

### Analysis of electrophysiological data

Recordings of electrophysiological data were taken from fifteen brainstem locations, and the digitized waveforms loaded into the MATLAB programming environment (The MathWorks, Natick, MA, USA). Because the time of contact varied slightly from trial-to-trial due to variations in the speed of the servo motor, trials were aligned based on the start of the physiological response. For each recording location and condition, the threshold for activity was taken to be 60% of the mean peak value of the local field potential waveform. The start of the physiological response was taken to be the time in each trial where the local field potential exceeded this threshold. For each trial, a 750 ms length of data was extracted, centered at the threshold crossing point. These data were then used to extract spike times for the units.

A MATLAB based, semi-supervised spike-sorting algorithm was used to identify the best-isolated single unit from each recording site. The sampled data were first digitally band-pass filtered between 300 and 3,000 Hz. Putative spike events were detected using a thresholding operation, and a window of data extracted around each event. Spike events were then clustered using peak height, peak width, trough depth, trough width, and the projection onto the first three principal components using a standard mixture-of-Gaussians model [33]. The clustering results were then visually inspected to select for the best isolated spike cluster, which was chosen as the target neuron for that recording. This cluster was used to generate a spike template for the best isolated spike.

All spike events were compared to this template and the least-squares error computed using a small window around the spike event. A threshold on the least-squares error determined which events were labeled as a spike from this unit. This threshold was manually determined for each unit and condition in order to balance detected spikes against noise events. Using this approach, 15 well-isolated units were found from 15 recording sites across six animals. Spike times and local field potential waveforms (the raw waveforms) were saved for further analysis.

### Analysis of spike times and local field potentials

Because the experiment could not provide absolute times of stimulus onset, it was necessary to estimate the contact duration from the spiking responses of each unit. For each unit, a peri-stimulus time histogram (PSTH) was formed using a bin width of 15 ms. The start of contact was taken to be the point at which the PSTH exceeded three times the mean firing rate. The end point of contact was defined as the point at which the PSTH returned below twice the mean firing rate. Spikes within this window were taken to be spikes in response to the stimulus, and spikes outside this window taken to be background spikes. Spike rates and response durations were calculated using this window. All statistical tests (two-sided t-tests, ANOVA) reported below were computed using the MATLAB statistics toolbox.

To classify the experimental condition from the recorded local field potential response, a maximum likelihood approach was tested. For each condition at each recording site, the broad-band LFP was pointwise-averaged to create a mean template waveform. Using a leave-one-out cross-validation approach, one trial was reserved for testing while the other 28 trials were averaged to create the template waveforms. The test trial was classified using a maximum likelihood approach with a multivariate Gaussian assumption [34]. Under these assumptions, the likelihood of the local field potential as an *N*-dimensional vector of voltages **v** in condition *j* was given by

$$\begin{array}{c} p\left(v|j\right)=\frac{1}{{\left(2\pi \right)}^{N/2}{\left|\Delta_{j}\right|}^{1/2}}\exp\left(-\frac{1}{2}{\left(v-\mu_{j}\right)}^{\prime }\Delta_{j}^{-1}\left(v-\mu_{j}\right)\right) \end{array}$$ (1)

where *μ*_*j*_ was the mean field-potential voltage, estimated over 28 training trials for condition *j*, which was an *N*-dimensional vector. The covariance matrix **Δ**_*j*_ was assumed to be diagonal with all entries given by $\sigma_{j}^{2}$. The quantity *σ*_*j*_ was estimated by taking the mean over time of the standard deviation of the voltage calculated at each time *t* using the data from the 28 trials. This form of the covariance assumed independence between each time sample and constant variance over time. Given a voltage trace recorded experimentally, classification was done by selecting the condition with the largest posterior likelihood, which was proportional to the likelihood times the prior for each condition *j*. Assuming equiprobable conditions, the decision rule was

$$\begin{array}{c} j^{*}={\text{argmax}}_{j}p\left(v|j\right) \end{array}$$ (2)

This was implemented using the log of the likelihood function, by finding the maximum over *j* of the expression $-0.5{\left(v-\mu_{j}\right)}^{\prime }\left(v-\mu_{j}\right)/\sigma_{j}^{2}-0.5\sum_{i=1}^{N}\ln\left(\sigma_{j}^{2}\right)$.

**Fig 2:**
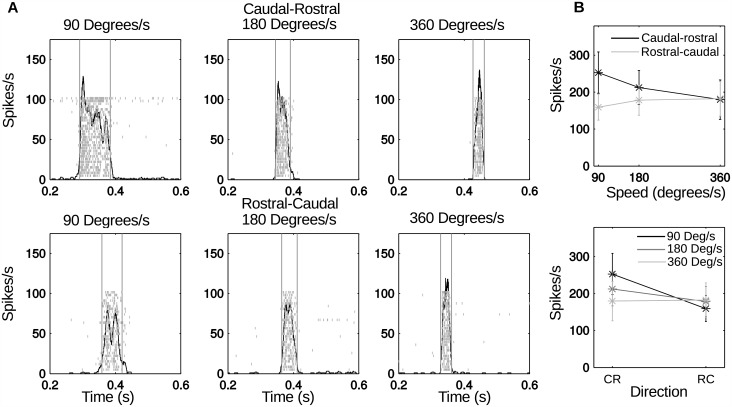
Response of one example neuron to stimulation at different speeds and directions. (A) Raster plots and PSTHs. The vertical lines indicate the contact window determined by thresholding the PSTH. The shifts in the response along the time axis between the different conditions are due to the differences in stimulus alignment between conditions. All PSTHs indicate a strong response to the stimulus and a relatively low baseline firing-rate. (B) Mean spike rate for each condition, with error bars indicating the standard deviation across all trials.

One additional complication, however, was that it was unreasonable to assume an absolute start time to the template in each trial. There was trial-to-trial variability in the start time, and, as can be seen in Fig 2, the responses for different conditions began at different absolute times. To compensate for the unknown start time, the template was compared to the test voltage trace at different shifts from 100 ms before to 100 ms after the stimulus, with a resolution of 2.5 ms. Classification was then done with the following rule

$$\begin{array}{c} j^{*}={\text{argmax}}_{j}{\text{argmax}}_{\tau }p\left(v_{t-\tau }|j\right) \end{array}$$ (3)

This created a template matching scheme which was invariant to shifts in the target template. This shift allowed for asynchronous classification of the waveform.

In order to classify the experimental condition based on the measured spike trains, a probabilistic model of the experimental spiking was used to compute the likelihood of each condition. Using the leave-one-out cross-validation approach on each trial, the PSTH was formed for each condition of each single unit response using 3 ms bins. The single unit PSTHs were normalized to a maximum value of 1, if at least one spike occurred in that bin in every trial, and to a minimum probability set by the average spontaneous spike rate. Each bin was modeled as an independent binary random variable with a probability of spiking given by the normalized PSTH. This resulted in a joint distribution for each condition. This approach assumed independent spike times, which were used to compute the posterior distribution of a stimulus [33]. Using this model, the test spike train was classified using a maximum likelihood approach. This provided an approximation of the encoding of stimulus conditions by individual spike trains.

The joint probability distribution of a set of spike times *t*_1_, *t*_2_, …, *t*_*n*_ over the interval *T* divided into *N* bins was then given by

$$\begin{array}{c} p\left(t_{1},t_{2},\ldots ,t_{n}|j\right)=\prod_{\left\{i|\phi (i)=1\right\}}p_{i}\prod_{\left\{i|1-\phi (i)=1\right\}}1-p_{i} \end{array}$$ (4)

where *ϕ*(*i*) is a function which has value 1 if for any spike *j*, (*i* − 1)*T*/*N* ≤ *t*_*j*_ < *iT*/*N* (otherwise zero), and *p*_*i*_ is the probability of spiking in the *i*th bin (derived from the normalized PSTH). To classify the experimental condition, the condition with the maximum likelihood was selected

$$\begin{array}{c} j^{*}={\text{argmax}}_{j}p\left(t_{1},t_{2},\ldots ,t_{n}|j\right) \end{array}$$ (5)

As with the local field potentials, it was not reasonable to assume that an absolute start time was known. It was therefore necessary to test over a range of time shifts in the experimental spike train. This gave a classification approach that does not rely on knowledge of an absolute start time. For time shifts *τ* ranging from 100 ms before to 100 ms after the start of stimulation (with a resolution of 2.5 ms), the probability of each condition given the shifted spike train was calculated. The maximum probability was taken as the probability for that class.

$$\begin{array}{c} j^{*}={\text{argmax}}_{j}{\text{argmax}}_{\tau }p\left(t_{1}-\tau ,t_{2}-\tau ,\ldots ,t_{n}-\tau |j\right) \end{array}$$ (6)

The classification schemes for the spike trains and local field potentials were used to decode the experimental condition from the recorded activity, yielding a measure of the potential to discriminate between experimental responses in different conditions. Higher classification rates indicated a larger difference in the experimental response. These methods estimate the lower bound on the optimal decoding performance, which may require more advanced statistical modeling and processing.

## Results

The present study was limited to recordings from multi-whisker-projection neurons from spinal trigeminal nuclei interpolaris and oralis. We begin by demonstrating that these neurons exhibit a central tendency to code for either stimulus direction, stimulus speed, or both, and then develop a lower bound on the performance of an optimal estimator for speed and direction based on the field potentials and spike trains of individual neurons. The relevant data for the figures presented in this section can be found in the S1–S5 Files.

### Examples of responses to varying stimulation speed and direction

The neural responses of the 15 units were analyzed to study the response of trigeminal brainstem neurons to variation in the speed and direction of stimulation. Fig 2 shows the response of a typical recorded neuron. As expected, the peri-stimulus time histograms (PSTHs) show a clear increase in the spike rate over the baseline activity for all conditions. At higher speeds the response becomes narrower in duration, consistent with the decrease in contact time (shown by the light grey vertical lines).

Different conditions also appear to show different patterns in the spiking activity. At slower speeds, there is an initial burst of spikes followed by a drop in spike rate, suggesting spike rate adaptation during contact. Six of the 15 units showed this spike rate adaptation for at least one condition. In addition, two units showed initial spiking at the start of stimulation and then spiking on release. To some degree, these two different types of adaptation may resemble the “slowly” and “rapidly” adapting responses observed in trigeminal ganglion neurons [28]. However, these adaptation characteristics were not observed in all stimulation conditions, and 7/15 neurons did not show any adaptation effect. Because all neurons in the present study responded to multiple whiskers they are likely to receive a complex combination of RA and SA afferents from different whiskers. It was therefore not possible to precisely classify adaptation characteristics, and these effects were not analyzed further.

Instead, we focus on change in spike rate related to speed and direction. Fig 2B shows these effects for the neural data shown in Fig 2A. The plots shows the mean spike rate with error bars indicating the standard deviation over the 29 trials. In one direction, the rate appears to increase with speed, while in the other the rate decreases. The slowest speed shows a significant difference in spike rate between the two directions.

### Spike rate is closely related to stimulus direction and to stimulus speed

**Fig 3:**
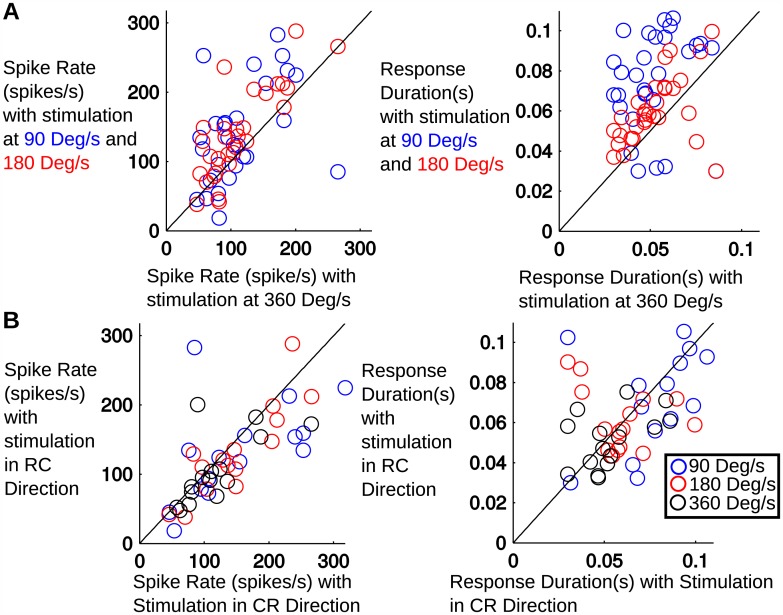
**(A) Comparison of the average spike rate and duration of the response during stimulation at 360°/*s* vs stimulation at 90°/*s* and 180°/*s***. There is a clear trend in increased response durations at 90°/*s* and 180°/*s*, with the 90°/*s* stimulation lasting the longest. There is a significant increase in spike rate in the 90°/*s* and 180°/*s* conditions when compared to the 360°/*s* condition. **(B) Comparison of spike rate and duration of the response during stimulation in the caudal-rostral direction vs stimulation in the rostral-caudal direction.** There is a statistically significant increase in spike rate in the caudal-rostral direction, but there is no significant change in the response duration.

Unsurprisingly, we found that for most neurons, response duration was increased for slower speed stimuli, but was unaffected by direction of stimulation. More surprising, however, is that neurons tended to slightly increase spike rate for slower speed stimuli. Fig 3A generalizes these effects over all neurons, comparing average spike rate and response duration for the slowest speed with the two higher speeds. Each point represents the average response of one unit. Responses to stimulation at both 90°/*s* as well as 180°/*s* were found to have significantly higher spike rates than responses to stimulation at 360°/*s*. (*p* = 0.026 and *p* = 0.0003, respectively, paired t-test). Thus on average, this group of neurons responded to slower stimuli with longer responses and slightly higher spike rates.

Strong effects were observed when we compared the response to the directionality of the stimulus. Fig 3B compares the average spike rate and response duration for the caudal-rostral (x-axis) and rostral-caudal (y-axis) directions for the 90°/*s*, 180°/*s* and 360°/*s*. Each point represents the average response of a single unit. The spike rate is significantly different (paired t-test, *p* = 0.04) between the two directions.

The tuning of the firing-rate of individual cells to speed and direction was further explored using an ANOVA test. The statistical comparisons of data for each cell were evaluated using ANOVA at the 95% confidence level (*a* = 0.05). The 3-factor ANOVA looked at main effects on firing rate and allowed for the evaluation of cells that were tuned to the contact phases, speed, and direction. The contact phases included pre-contact, contact, and post-contact. Contact was defined using the thresholds described previously (shown in Fig 2A, vertical lines). Analyzing the spike rates, 80% of the units had higher rates for stimulation in the caudal to rostral direction than in the rostral to caudal direction. Comparing the spike rates at 90°/*s* to 360°/*s*, 67% of units showed higher rates for the slower stimulus speed. We found that all of the cells (100%) showed a significant (*p* < 0.05) change in firing rate during contact, indicating that they were involved in the sensory response. The majority of cells (73%) were tuned for direction and 83% of cells were tuned for speed. Over half of the population (66%) was tuned for both speed and direction, and all cells were tuned for at least one of these variables. As a population the average firing rates were slightly higher for caudal-rostral stimulation and tended to decrease as movement speed increased, although the activity of individual cells exhibited a variety of relationships with the stimulus types.

### Multi-unit activity is more strongly tuned for speed and direction than single unit spike rates

As described above, the analysis of single unit spike rates showed a significant variation with speed and direction in 66% of units. Although this suggests that spike rate is modulated by the experimental variables, not all neurons show a significant response to both conditions. It is possible that some neurons are sensitive to direction of the stimulus and others more sensitive to the speed.

To improve understanding of how these variables are encoded by the population of neurons in the trigeminal nuclei, we examined the variation in multi-unit activity across multiple frequency bands of the multi-unit response. The recorded broad-band neural signals were filtered into different narrow bands using a filterbank of bandpass filters with different bandwidths and the signal envelope in each band was computed. Using the average signal envelope amplitude in each phase of the trial, the ANOVA procedure above was run on the average power for each band (with *p* < 0.05 as the criterion for significance).

**Fig 4:**
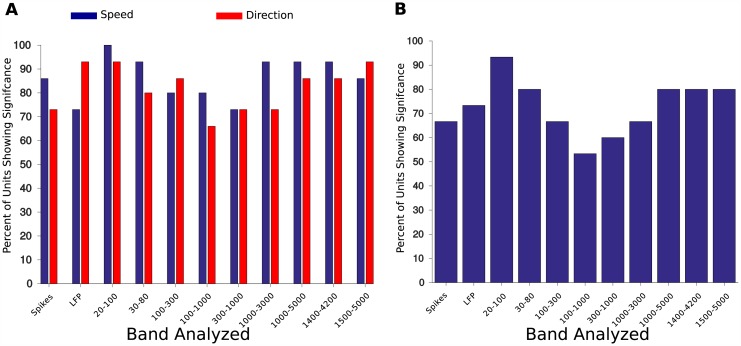
Percentage of recording sites with a significant tuning to speed, direction, or both as determined by an ANOVA analysis of signal power in different frequency bands. Average power in each band was computed from the output from a bank of bandpass filters and significance levels were set at *p* < 0.05. (A) Percentage of recording sites with a significant tuning to speed or direction. Several bands driven by multi-unit activity (1,000–3,000 Hz, 1,000–5,000 Hz) are sensitive to both speed and direction. (B) Percentage of recording sites with significant tuning to both speed and direction. In several bands driven by multi-unit activity (1,000–3,000 Hz, 1,000–5,000 Hz) more than 80% of recording sites are sensitive to both speed and direction. In contrast, 66% of single unit spike rates were sensitive to both conditions.

The results of this analysis are shown in Fig 4. Fig 4A shows the percentage of units recorded which show significant differences as a function of speed or direction. This percentage is shown for the single unit spike rate and also for different frequency bands of the LFP (some overlapping). A majority of units in all conditions were sensitive to speed or direction. Sensitivity to speed was higher in five LFP bands than in the single unit spike rate. In many units, the spike rate was significantly different as a function of direction. The LFPs bands which had the lowest percentage of units sensitive to direction were between 100 and 3000Hz.

Interestingly, over 80% of units are sensitive to both speed and direction when measured over bands containing large amounts of multi-unit activity (1000–3000 Hz, 1500–4200 Hz). Broadly, all frequency bands have a higher percentage of units which are sensitive to the conditions than the single unit spike rates. Interpreting the broad-band local field potential as a correlate of population activity, this suggests a robust population representation of speed and direction in the trigeminal nuclei.

In summary, the change in the power of the envelope of the multi-unit activity is, in general, significantly different for different stimulus conditions (speed and direction). We therefore next investigated the possibility of decoding experimental condition from the local field potentials and spike trains recorded experimentally.

### Local field potentials enable more accurate classification of speed and direction than single unit spike trains

To further test the encoding of speed and direction in these brainstem neurons, we considered the problem of classifying, or decoding, the experimental condition from the local field potentials and spike trains recorded experimentally. This analysis represents a lower bound on the performance of an optimal estimator of speed and direction based on the field potentials or spike trains of individual neurons.

**Fig 5:**
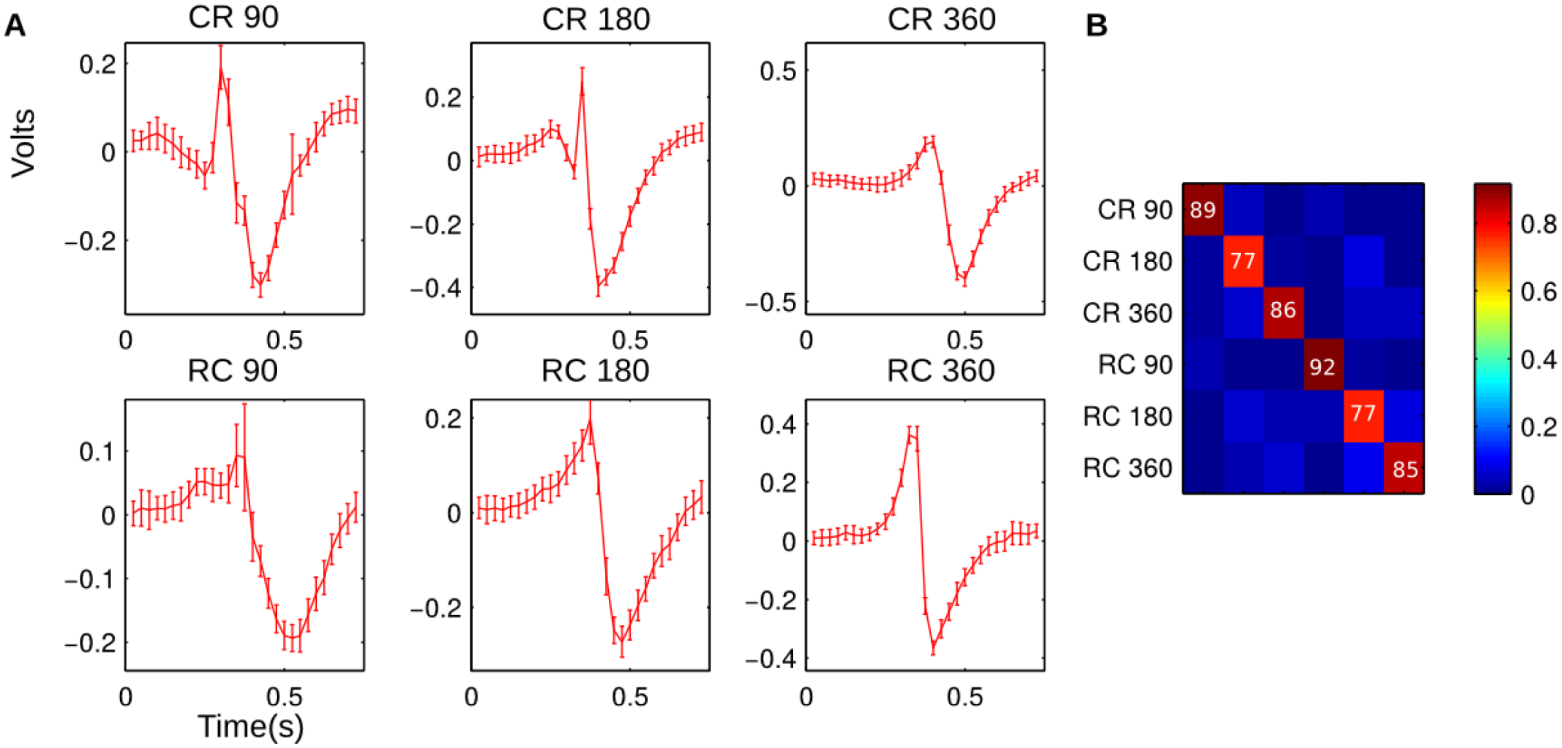
This figure shows the result of a template-matching classification method using the broad-band local field potentials. Using a cross-validation approach for each neuron, the training data (consisting of the raw microelectrode recording filtered between 1 and 10000 Hz) was used to estimate a template for each experimental condition. The test data were then compared to each template and the log likelihood calculated. To make classification asynchronous, the template was tested at different lags and the lowest error selected for that condition. The experimental condition with the lowest difference was used to classify the test data. Panel A shows the template waveforms for an example recording site. The templates are very distinct, with the standard deviation showing a relatively low trial-to-trial variability. Panel B shows the average confusion matrix for the template classification. The numbers in Panel B are the per-condition probability of correct classification.

Importantly, this analysis does not imply that these methods are used by the nervous system. Rather, the approach simply analyzes how distinct the neural responses are in the experimental conditions. This analysis was performed on the broadband local field potentials using the maximum likelihood classifiers defined in Materials and Methods using a leave-one-out cross validation approach. Fig 5A shows the mean local field potentials and standard deviation estimated over the 29 trials at a single recording site. These templates are used for the maximum likelihood classification. As can be seen, there is a distinct template waveform for each condition. Fig 5B shows the confusion matrix averaged over all recording sites. The average probability of correctly identifying the experimental condition was 84%. The distinct template waveforms for the LFPs result in high classification rates. Assuming this activity reflects the overall population activity, this suggests a robust population encoding.

**Fig 6:**
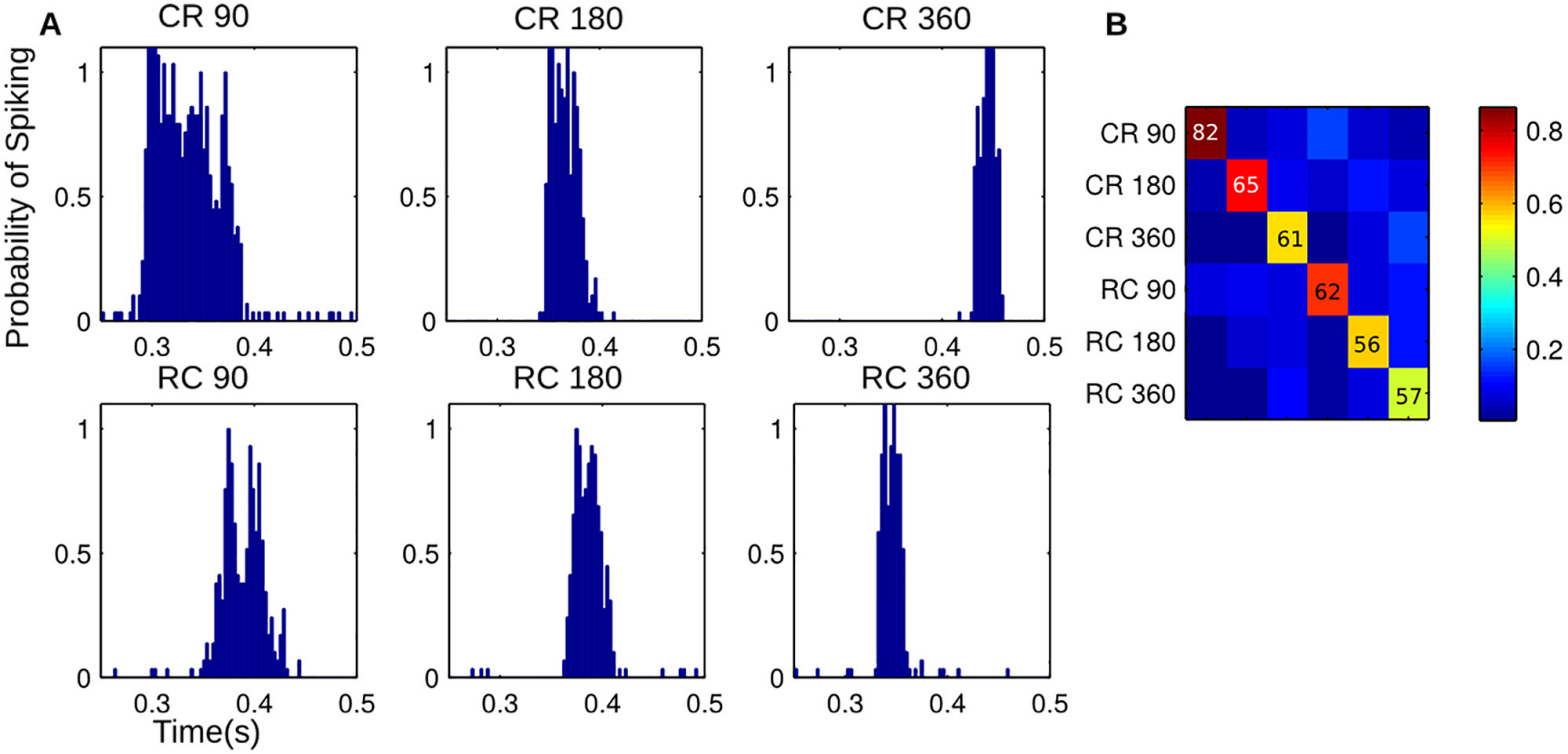
This figure shows the results of a classification scheme using the spike train from a single unit. Using a cross-validation approach, the training data for each unit was used to calculate the PSTH. This was normalized to get the probability of firing a spike in each bin. For the test spike train, the probability of observing the spike train given each condition was calculated, assuming independent probabilities of spiking in each time bin. This was repeated for different lags and the highest probability selected for that condition. The condition with the highest probability was used to classify the test spike train (a maximum likelihood approach). Panel A shows the normalized PSTHs for an example unit. Panel B shows the average confusion matrix over all units. The average probability of correct classification is lower than when using the LFPs. Although not necessarily indicative of how the neural system decodes these neural responses, these results, along with Fig 5, suggest that the population response is more robust than the response of a single unit.

The maximum likelihood classification with leave-one-out cross-validation was also applied to the single unit spike trains to determine the experimental condition. The normalized PSTH was interpreted as the probability of firing a spike in a particular time bin of the response. This approach represents a lower bound on the performance of an optimal detector that takes into account the statistics of the stimulus conditions and the statistical relationship between spike times. Fig 6A shows an example PSTH for each experimental condition from a single unit. Fig 6B shows the confusion matrix for classification, averaged over all units. The average probability of correctly identifying the experimental condition was 64% (well above the 16.67% chance level for six classes). Classification rates seem to be highest for the lowest speeds, which may be related to a larger total number of spikes in each trial.

Interestingly, the classification using the LFP is more reliable, likely due to conditions which are more easily discriminated. Comparing the probability of correct classification over the 15 recording sites, the probability of successful classification using the LFP was found to be significantly higher than the probability of correct classification using the spike trains (Wilcoxon rank-sum test, *p* = 0.0021 < 0.05). Assuming that the LFP is the reflection of the aggregate activity of the population of neurons in this region of the brainstem, it suggests that the population response is very robust. On the other hand, the lower classification rate for an individual spike train suggests that spike trains have higher trial-to-trial variability.

## Discussion

### Cell types in the trigeminal nuclei

Together, spinal trigeminal nuclei oralis and interpolaris are important stations in processing information arriving from the trigeminal ganglion through to the cerebellum, superior colliculus, thalamus and ultimately sensory cortex [20, 30, 31, 35, 36]. Previous studies of sensory information processing within the trigeminal nuclei have focused on receptive field size [14, 16, 30] and the physiology of angular tuning in projection and local circuit neurons [31, 37, 38].

Although much remains to be understood about processing in the trigeminal nuclei, it is well established that single whisker receptive fields are associated with local circuit neurons [15, 27, 30, 39], while multi-whisker receptive fields are associated with neurons that project to more central structures [27, 39, 40]. As yet, however, there is little understanding of the role of multi-whisker receptive fields, which are a defining feature of these projection neurons. The experiments of the present work were therefore designed to investigate the responses of neurons in the trigeminal brainstem to tactile stimulation of the entire whisker array.

### Angular tuning vs. direction tuning

Responses of primary sensory neurons in the trigeminal ganglion are known to exhibit strong angular tuning [4, 6, 9, 28, 29], and this tuning is also found within the trigeminal nuclei [31, 32]. Specifically, the receptive fields of neurons in rostral interpolaris exhibit angular tuning for upward deflections, while neurons in the caudal regions do not exhibit such tuning bias [31]. Intriguingly, one recent study tested the consistency of angular tuning across primary and secondary somatosensory cortical areas, as well as ventroposterior medial thalamus and superior colliculus [38]. Results showed that in all regions a small number of neurons display consistent angular tuning for at least some vibrissae [38]. An unresolved question, then, is how angular tuning is transformed into directional tuning, in which a neuron exhibits selectivity for stimulus motion in a particular direction across multiple whiskers.

Multi-whisker receptive fields in interpolaris can be explained by the spatial extent of the dendritic trees of projection neurons [27]. Although little is known about how trigeminal neurons interconnect to create functional neural circuits, it is likely that directional tuning can be achieved through lateral inhibition. While there is no clear evidence for lateral inhibition in SpVi, PrV circuits show lateral inhibition of the principal whisker by adjacent whiskers [41, 42]. Similar circuitry may also operate within regions of oralis and interpolaris. This type of computation may be particularly important given that SpVi projection neurons with large multi-whisker receptive fields excite higher-order midbrain and diencephalic cells thought to mediate head orientation towards an externally generated vibrissa deflection [20].

A second possible mechanism for directional tuning lies in the mechanical response of the vibrissae to rapid directional deflections. The intrinsic curvature of the vibrissae [43–46] ensures that both bending and vibrations will be much larger when stimulation is delivered from caudal to rostral instead of from rostral to caudal. Although angular tuning at the level of a single whisker offers high spatial resolution, integration of sensory information across multiple whiskers provides more robust information about contact as well as the ability to localize towards a moving object [47–51].

### Population codes for speed and direction

The results presented here suggest that as a population, the spike rate of trigeminal brainstem neurons encodes information about both stimulus direction and speed. In general, more cells were tuned for speed, but many cells were tuned to direction as well, and more than half the population was tuned to both variables. The results of decoding the stimulus condition with the single unit responses and the LFPs have interesting implications for population encoding in these structures. Single unit classification was well above chance, but worse than decoding using the LFPs. The LFPs have low trial-to-trial variability and a classification rate of around 90%. These results indicate that the population provides a more reliable estimate of these variables than individual units, and that while trial-to-trial variability in spikes is fairly high, redundancy helps encode physical parameters reliably.

Summarizing, these brainstem regions appear to contain a robust population-level representation of speed and direction of a real object moving through the whisker field. As in cortical regions [52–58], local field potentials and multi-unit activity may be sufficient for stimulation and decoding within trigeminal sensory nuclei. Further studies of single unit decoding in the trigeminal nuclei are still warranted, however, given that individual spike trains are likely to be encoding additional features of the stimulus, such as relative angles or surface texture information.

### Potential behavioral significance

Previous work in the head-fixed animal has demonstrated that responses in SpVi are minimal during non-contact whisking [59, 60]. In view of the present finding that trigeminal brainstem neurons respond to directed motion over multiple whiskers, we predict that these neurons will respond most strongly to coherent motion of a tactile stimulus across the array. This type of stimulation could be generated by grooming behavior (sweeps of the forepaws across the face), by moving prey, or by a conspecific [61, 62]. Thus we suggest that these brain regions are likely to be involved in detecting, tracking, and orienting to an external moving stimulus.

Detecting and orienting behaviors generally must be executed quickly. SpVi could mediate these movements via fast reflex loops through the facial motor nucleus [63], simultaneously modulating attention and alertness through its projections to the laterodorsal tegmental nucleus and pedunculopontine tegmental nucleus [64]. SpVi also projects to the superior colliculus and has extensive reciprocal projections to the cervical spine [65, 66], consistent with a role in whisker-based control of head orientation and position. With the assumption that following along a wall or floor could often generate directionally-coherent motion across the array [67, 68], we suggest that SpVi may specifically contribute to keeping the head in a particular posture relative to these surfaces.

The placement of SpVi within the larger context of the trigemino-thalamocortical system suggests that it could also play an important role in allowing the rat to determine if an object is moving within the array, even while the whiskers themselves are actively moving. The reasoning here is as follows: in addition to its extensive involvement in brainstem reflex loops, SpVi is the start of the paralemniscal pathway, which projects through the posteromedial nucleus of the thalamus (PoM) to primary somatosensory cortex [64]. PoM is constitutively inhibited by zona incerta (ZI), but ZI is itself inhibited by primary motor cortex (M1). Thus information transmission from SpVi to cortex is gated by activity in M1 [69]. This circuitry suggests that whisking commands sent from M1 could inhibit ZI, potentially releasing PoM from inhibition and allowing activity from SpVi to reach primary somatosensory cortex.

Once information from SpVi is allowed through PoM, it is sent to layers 5a and 1 of primary somatosensory cortex. This information is likely processed by layer 5 (L5) pyramidal neurons, which have dendrites in both layer 1 (L1) and L5. Intriguingly, concurrent input to the L1 and L5 dendrites of these neurons has been implicated in sensory feature association [70]. The proposed mechanism is that the apical tufts of L5 pyramidal neurons harbor an initiation zone for calcium-mediated plateau potentials triggered by concurrent input to the L5 and L1 compartments. These calcium spikes can cause whole-cell depolarization, potentially allowing these cells to become more responsive to subsequent inputs to either compartment [70]. This mechanism would allow paralemniscal inputs to upregulate L5 cells and thereby modulate the output of the cortical column. In this manner, the paralemniscal pathway may modulate the output of cortex with information specifically about externally-generated touch during active touch. This mechanism would allow the perception of a mismatch between the expected input (generated by active touch alone), compared with the actual input that combines both active touch and exogenous movement.

In the context of established trigeminal circuit organization, the present finding that SpVi encodes both direction and speed of an external stimulus predicts the following: during passive touch, when a rat is not whisking and encounters movement across the whisker array, information from SpVi is not allowed through the paralemniscal pathway (through PoM) to cortex. Passive tactile information is sent through the lemniscal pathway, from PrV to VPM to primary somatosensory cortex. At the same time SpVi sends passive touch information through short-latency circuits to spinal/hindbrain/midbrain centers, allowing orienting behaviors.

In contrast, when a rat is actively whisking and encounters movement within the whisker array, the information is processed not only though the lemniscal and short-latency reflex systems, but is also processed in parallel by the paralemniscal pathway through PoM to cortex, where it may upregulate the output of L5 pyramidal cells, allowing the rat to disambiguate the sensation of touch due to internally vs. externally generated movement. SpVi may therefore allow the rat to more clearly perceive the sensory stimuli generated by moving external objects, even during active touch.

## Supporting Information

S1 File Spreadsheet of Data for Fig 2. This file contains the data used in Fig 2. There is one sheet for each of the six experimental conditions. The spike times for these conditions are listed for each trial in seconds. These were used to create the histograms seen in Panel A. The times for the contact window (vertical grey lines) are also given. A final sheet gives the rates and durations shown in Panel B.
(XLSX)

S2 File Spreadsheet of Data for Fig 3. The file contains the rate (in spikes/s) and duration (in s) for all units shown in Fig 3. Each experimental condition has a separate rate and duration included.
(XLSX)

S3 File Spreadsheet of Data for Fig 4. This contains all the data used in the ANOVA analysis of the spike rates, the LFPs, and the individual LFP bands shown in Fig 4. There is one sheet for each frequency band, one for the LFP, and one for the spike rate. Each sheet contains the results of the ANOVA analysis, showing significance for speed of stimulation, direction of stimulation, and both variables. All of the data used in the analysis is then given, either the spike rate in spikes/s, the RMS value of the LFP, or the RMS values of the LFP band.
(XLS)

S4 File Spreadsheet of Data for Fig 5. This spreadsheet shows the data displayed in Fig 5. The average confusion matrix, along with the confusion matrices for each individual unit is given.
(XLSX)

S5 File Spreadsheet of Data for Fig 6. This spreadsheet shows the data displayed in Fig 6. The average confusion matrix, along with the confusion matrices for each individual unit is given.
(XLSX)
